# Long-term outcomes of early posterior scleral contraction for myopic macular retinoschisis in patients up to 30 years

**DOI:** 10.3389/fmed.2026.1892373

**Published:** 2026-08-31

**Authors:** Linyan Zheng, Jian Zhao, Caiyu Yu, Liang Dong, Anquan Xue, Shuangqian Zhu

**Affiliations:** 1 National Clinical Research Center for Ocular Diseases, Eye Hospital, Wenzhou Medical University, Wenzhou, China; 2 National Engineering Research Center of Ophthalmology and Optometry, Eye Hospital, Wenzhou Medical University, Wenzhou, China

**Keywords:** high myopia, macular retinoschisis, pathologic myopia, posterior scleral contraction, prevention

## Abstract

**Aim:**

To evaluate the long-term anatomical and functional outcomes of early posterior scleral contraction (PSC) in patients up to 30 years with myopic macular retinoschisis (MRS).

**Methods:**

This retrospective study included 21 patients (27 myopic eyes) with MRS, with or without macular detachment, who underwent PSC and had a postoperative follow-up exceeding 2 years (*n* = 27). Eyes with MRS underwent PSC, an extraocular procedure involving the placement of a genipin-crosslinked allogeneic strip at the posterior pole to shorten axial length and relieve vitreoretinal traction. Anatomical resolution of MRS, best-corrected visual acuity (BCVA), axial length change, were analyzed in patients with approximately 5-year follow-up records (the 13-eye main analysis cohort); while the overall safety profile was reported using the 27-eye descriptive cohort.

**Results:**

At approximately postoperative year 5, complete resolution of MRS was achieved in 4 eyes (30.8%, 95% CI: 12.7%−57.6%), and partial resolution occurred in 9 eyes (69.2%, 95% CI: 42.4%−87.3%); BCVA did not improve significantly compared to the baseline level (*P* = 0.482, *n* = 13). Mean axial length shortened significantly immediately postoperatively (*P* < 0.001, *n* = 13) but gradually regressed to near baseline at approximately postoperative year 5 (*P* = 0.183, *n* = 12). In the 27-eye descriptive cohort, no eye progressed to macular hole or retinal detachment, and metamorphopsia all resolved within 8 months. No sight-threatening intra- or postoperative complications, such as cataract, iatrogenic macular hole, were observed.

**Conclusion:**

Early PSC may offer favorable anatomical stability and acceptable safety in young MRS patients. However, larger prospective controlled studies are needed to confirm its long-term efficacy and safety.

## 1 Introduction

Since the advent of optical coherence tomography (OCT), myopic traction maculopathy (MTM) has become readily detectable. Myopic macular retinoschisis (MRS) has emerged as one of the most common macular complications of high myopia, with an estimated prevalence ranging from 8% to 34% (1). MRS is a progressive condition, with studies indicating that 0.9% to 33.0% of cases may progress to a full-thickness macular hole and 3.4% to 37.5% to foveal detachment (2). Patients with MRS alone are often managed with clinical observation, because there is general agreement that vitrectomy in this population lacks a favorable risk-benefit profile. Vitrectomy is typically recommended only when there is significant vision loss or at high risk of progression (3, 4).

In young patients, MRS presents greater therapeutic challenges, as it is often associated with ongoing axial elongation and scleral expansion. While vitrectomy is effective for many progressing cases, treatment failure does occur, such as full-thickness macular hole or macular hole retinal detachment (5–9). In younger patients where the lens is typically preserved, postoperative cataract development is remarkably common. In fact, the proportion of patients who required phacoemulsification with intraocular lens implantation after vitrectomy was significantly higher than after posterior scleral contraction (PSC) (78.2% vs. 23.9%) (10). Moreover, vitrectomy cannot slow down axial elongation, which contributes to high recurrence rates and potential atrophy maculopathy (11). Thus, vitrectomy is a suboptimal solution for younger patients with MRS.

Given the often unsatisfactory visual prognosis of advanced MTM (12), there is a rationale for considering alternative early interventions in young patients with MRS and clear lenses, prior to irreversible vision impairment. Our prior work demonstrated the feasibility of PSC in MRS. However, those studies were not focused on patients up to 30 years and had limited follow-up (13, 14). Evidence supporting early PSC in younger patients with MRS remains scarce. Therefore, this study aimed to evaluate the long-term anatomical and functional outcomes of prophylactic PSC in a cohort of patients up to 30 years with MRS.

## 2 Materials and methods

### 2.1 Study participants

From our dedicated registry of patients who underwent PSC, we identified and enrolled patients according to the following criteria. Inclusion criteria were patients up to 30 years old who underwent PSC for MRS, with or without macular detachment, between 2008 and 2019 and had a postoperative follow-up exceeding 2 years. In this study, early PSC was defined as surgical intervention performed at the stage of MRS (with or without macular detachment), before the development of a full-thickness macular hole or macular hole retinal detachment. Surgical indications for PSC in young adults included progressive OCT changes, visual decline, metamorphopsia, or prophylactic considerations. Patients were excluded if they had incomplete medical records or a shorter follow-up period, a history of other ocular conditions affecting the macula or optic nerve (e.g., choroidal neovascularization, glaucoma, etc.), prior vitrectomy for other indications, or severe systemic conditions considered inappropriate. Informed consent was obtained from all patients prior to surgery. The study protocol adhered to the tenets of the Declaration of Helsinki. Ethical approval was granted by the institutional Ethics Committee.

### 2.2 General examinations

We collected preoperative data including best-corrected visual acuity (BCVA), manifest refraction spherical equivalent, axial length, intraocular pressure, macular OCT scans, anterior segment examination, and dilated fundus examination. All eyes underwent repeated examinations during follow-up, which included OCT imaging, axial length measurement, visual acuity tests, among others. The scheduled intervals were 1 week, 3 months, 6 months, and annually thereafter. BCVA was measured using the Chinese standard logarithmic visual acuity chart, converted to logarithm of the minimum angle of resolution (logMAR) format for data analysis, and Snellen for demonstration (Supplementary Table 1). Finger counting and hand motion were assigned values of 2.00 and 3.00 logMAR, respectively. Axial length was measured by partial coherence laser interferometry (IOLMaster, Carl Zeiss Meditec, Oberkochen, Baden-Württemberg, Germany). OCT was performed using a Stratus OCT (Carl Zeiss Meditec, Jena, Germany) or an Optovue OCT (Optovue, Inc., Fremont, CA, USA) to assess macular status. Visual and anatomic outcome, and complications of PSC for MRS were also analyzed. The primary outcome measures were (1) anatomical resolution of MRS on OCT and (2) improvement in BCVA. Secondary outcomes included axial length change, complication rates, and overall safety assessment.

### 2.3 Surgical procedure

All procedures were performed under general anesthesia by the same surgeon (AQX). The surgical technique has been described in detail elsewhere (13, 15, 16). Briefly, a three-quadrant peritomy was performed followed by blunt posterior dissection under general anesthesia. The eye was rotated superonasally using traction sutures under the lateral and inferior rectus muscles. After blunt dissection of the inferior oblique muscle for exposure, a customized, spindle-shaped genipin-crosslinked allogeneic strip was positioned at the posterior pole between this muscle and the optic nerve. Its inferior end was sutured 2–3 mm posterior to the rectus muscle insertion, avoiding vortex veins; the superior end was placed lateral to the superior rectus muscle. Following anterior chamber paracentesis and tension confirmation via finger measurement, ensuring no compression of vortex veins or the optic nerve, the strip was trimmed, tightened, and its superior end secured. Finally, the conjunctiva was closed with 8–0 sutures. We reassessed axial length and performed OCT on the day of surgery or postoperative day 1. By comparing these results with preoperative measurements, patients with insufficient axial length reduction underwent secondary scleral strip adjustment under local anesthesia to achieve further shortening. In P2, due to retinal detachment extending beyond the vascular arcades, PSC was combined with intravitreal air tamponade. Given the patient's youth and the known risks of vitrectomy (e.g., cataract, proliferative vitreoretinopathy), we adopted a stepwise approach: PSC first, with vitrectomy considered only if the condition failed to respond.

The evaluation of macular reattachment postoperatively utilized a radial scan protocol via OCT. Complete resolution was defined as resolution of MRS across all meridians; partial resolution was defined as persistent MRS in one or more meridians.

### 2.4 Statistical analyses

Statistical analyses were performed using SPSS software (version 26.0). Continuous variables are presented as mean ± standard deviation and range, BCVA is further presented with median and interquartile range (IQR). Generalized estimating equations with an exchangeable correlation structure were used to account for inter-eye and repeated-measure correlations. A linear model with identity link function was applied for both BCVA and axial length, with each entered as the dependent variable. The model included patient ID as the subject variable, eye laterality and follow-up time as within-subject variables, and follow-up time was entered as a factor (baseline = 0, 1 week postop = 1, approximately 5 years postop = 2). Multiple comparisons were adjusted with the Bonferroni method. Robust standard errors were used for parameter estimates. Sensitivity analyses were further performed by including only the right eye in patients with bilateral data, and by excluding those with additional surgeries (air tamponade, scleral-strip adjustment, and phakic intraocular lens implantation). Missing data were not imputed; analyses were based on available data only. Proportions for MRS resolution were computed with 95% confidence intervals using the Wilson score method, and single-eye sensitivity analysis was performed by including only the right eye in patients with bilateral data. A *P-value* of < 0.05 was considered statistically significant.

## 3 Results

Medical records of patients who underwent PSC at our hospital (*n* = 11,401) were reviewed. After applying the inclusion and exclusion criteria, 21 patients (27 eyes) were included in this study. To account for the heterogeneity in postoperative follow-up, 13 eyes (10 patients, highlighted in red in Supplementary Table 1) with documented records at approximately 5 years post-PSC (within ± 1.25 years) were used for further statistical analyses, the remaining eyes did not have a measurement within this window because of variable follow-up attendance in this retrospective real-world cohort. However, the overall 27 eyes were still presented, for descriptive purposes (Supplementary Table 1). The mean baseline age was 21 ± 4 years (range: 14 to 26 years). Baseline axial length was 28.81 ± 1.72 mm (range: 25.79–31.61 mm, *n* = 13), baseline BCVA was 0.10 ± 0.11 (median, IQR: 0.10, 0–0.22; logMAR; *n* = 13), and baseline spherical equivalent was −12.69 ± 3.64 D (range: −8.75 to −21.00 D, *n* =13). OCT showed MRS in all 13 eyes, involving the fovea in 5 eyes and sparing the fovea in 8 eyes. The mean postoperative follow-up period was 61 ± 8 months (range: 45–75 months, *n* = 13). P2 was phakic with an iris-claw intraocular lens at baseline. Concomitant macular detachment was present in one eye (P1). One eye (P2) exhibited both macular and peripheral retinal detachment, PSC was combined with intravitreal air tamponade in this case. P1 and P2 belonged to the 27-eye descriptive cohort.

At approximately postoperative year 5, complete resolution of MRS was achieved in 4 eyes (30.8%, 95% CI: 12.7%−57.6%), occurring gradually postoperatively. Partial resolution occurred in 9 eyes (69.2%, 95% CI: 42.4%−87.3%). In the sensitivity analysis (*n* = 10), complete resolution of MRS was observed in 4 eyes (40.0%, 95% CI: 16.8%−68.7%), partial resolution in 6 eyes (60.0%, 95% CI: 31.3%−83.2%); and these results were broadly consistent with the main analysis. Macular detachment was fully resolved in the 2 affected eyes. BCVA improved from 0.10 ± 0.11 (median, IQR: 0.10, 0–0.22; logMAR; *n* =13) to 0.08 ± 0.10 (median, IQR: 0, 0–0.22; *n* =13), this difference was not statistically significant (*P* = 0.482; 95% Wald CI for difference: −0.082 to 0.039). The single-eye sensitivity analysis (*n* = 10) remained statistically non-significant (*P* = 0.199). Sensitivity analysis (*n* = 9) excluding additional surgeries yielded similar results (*P* = 0.050). However, in terms of all 27 eyes, BCVA improved by 1 line in 5 eyes, by 2 lines in 3 eyes, by 4 lines in 1 eye, and improved from hand motion to 20/100 in 1 eye; BCVA remained stable in 14 eyes, decreased by 1 line in 1 eye, and by 2 lines in 2 eyes (Supplementary Table 1).

Following PSC, the mean axial length reduced from a preoperative 28.81 ± 1.72 mm (*n* = 13) to 27.39 ± 1.64 mm at 1 week postoperatively (*n* = 13) (*P* < 0.001; 95% Wald CI for difference: −1.811 to −1.018). The single-eye sensitivity analysis (*n* = 10) remained statistically significant (*P* < 0.001). Sensitivity analysis (*n* = 9) excluding additional surgeries yielded similar results (*P* < 0.001). Subsequently, axial length gradually regressed over time toward preoperative levels. At the approximately 5-year follow-up visit, the mean axial length (*n* = 12) showed no statistically significant difference but remained shorter, compared to the preoperative level (*P* = 0.183; 95% Wald CI for difference: −0.597 to 0.073). The single-eye sensitivity analysis (*n* = 9) remained statistically non-significant (*P* = 0.125). Sensitivity analysis (*n* = 8) excluding additional surgeries yielded similar results (*P* = 0.285).

During follow-up, five patients (P6, P7, P8, P16, and P17; 6 eyes; see Supplementary Table 1) underwent phakic intraocular lens implantation to correct refractive error after resolution of MRS. In two patients (P19 and P21), axial length reduction was deemed insufficient, prompting a secondary scleral strip adjustment.

No intraoperative complications were observed in all cases (*n* = 27). Metamorphopsia occurred in 12 of 27 eyes during the first postoperative week. Complete resolution was observed in 10 eyes by 4 months, in 1 eye by 6 months, and in the remaining eye by 8 months, with no lasting clinical sequelae. Besides, no patient developed subretinal hemorrhage, choroidal hemorrhage, choroidal neovascularization, retinal detachment, etc. Furthermore, there were no cases of scleral perforation, or migration of scleral strip. No iatrogenic macular holes, progression of MRS to macular hole, cataract, ocular hypotony or hypertension were documented.

**Figure 1:**
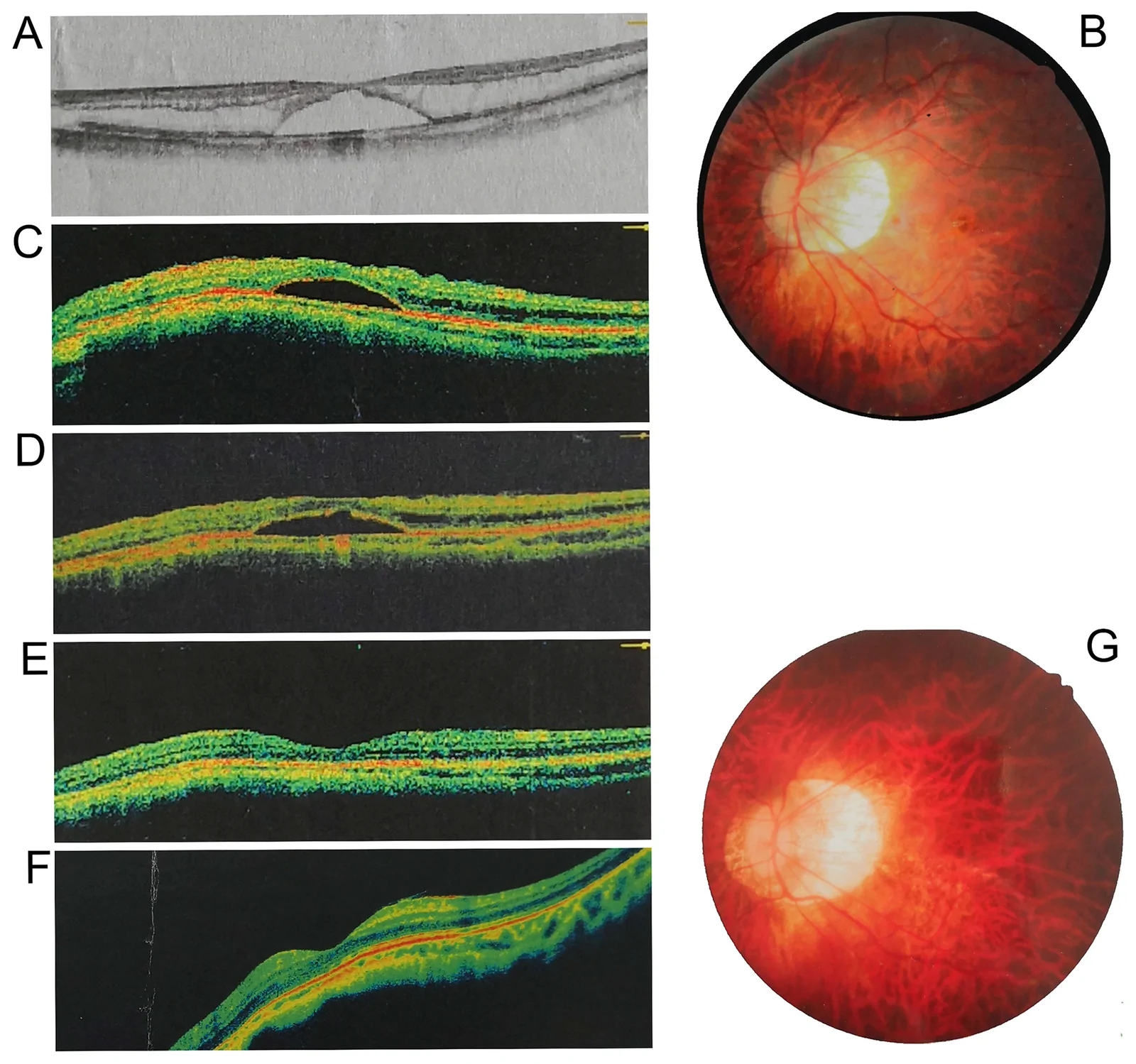
Gradual resolution of macular retinoschisis (MRS) and macular detachment (MD) following posterior scleral contraction (PSC) in a 20-year-old high myopia male (P1). **(A)** Preoperative OCT demonstrates apparent MRS and MD; **(B)** Corresponding preoperative fundus photograph shows diffuse chorioretinal atrophy and temporal crescent; **(C)** One week postoperative OCT reveals partial resolution of MRS and MD with retinal surface folds; **(D)** At 2 months postoperative, OCT shows further resolution of MRS and MD, with disappearance of retinal folds; **(E)** At 6 months postoperative, OCT confirms complete anatomical reattachment; **(F)** At 5 years postoperative, OCT demonstrates maintained structural stability; **(G)** Corresponding 5-year postoperative fundus photograph shows mild enlargement of temporal crescent compared to preoperative state. The patient's axial length was 31.64 mm preoperatively and 29.39 mm at 1 week postoperatively, representing a reduction of 2.25 mm following PSC.

**Figure 2:**
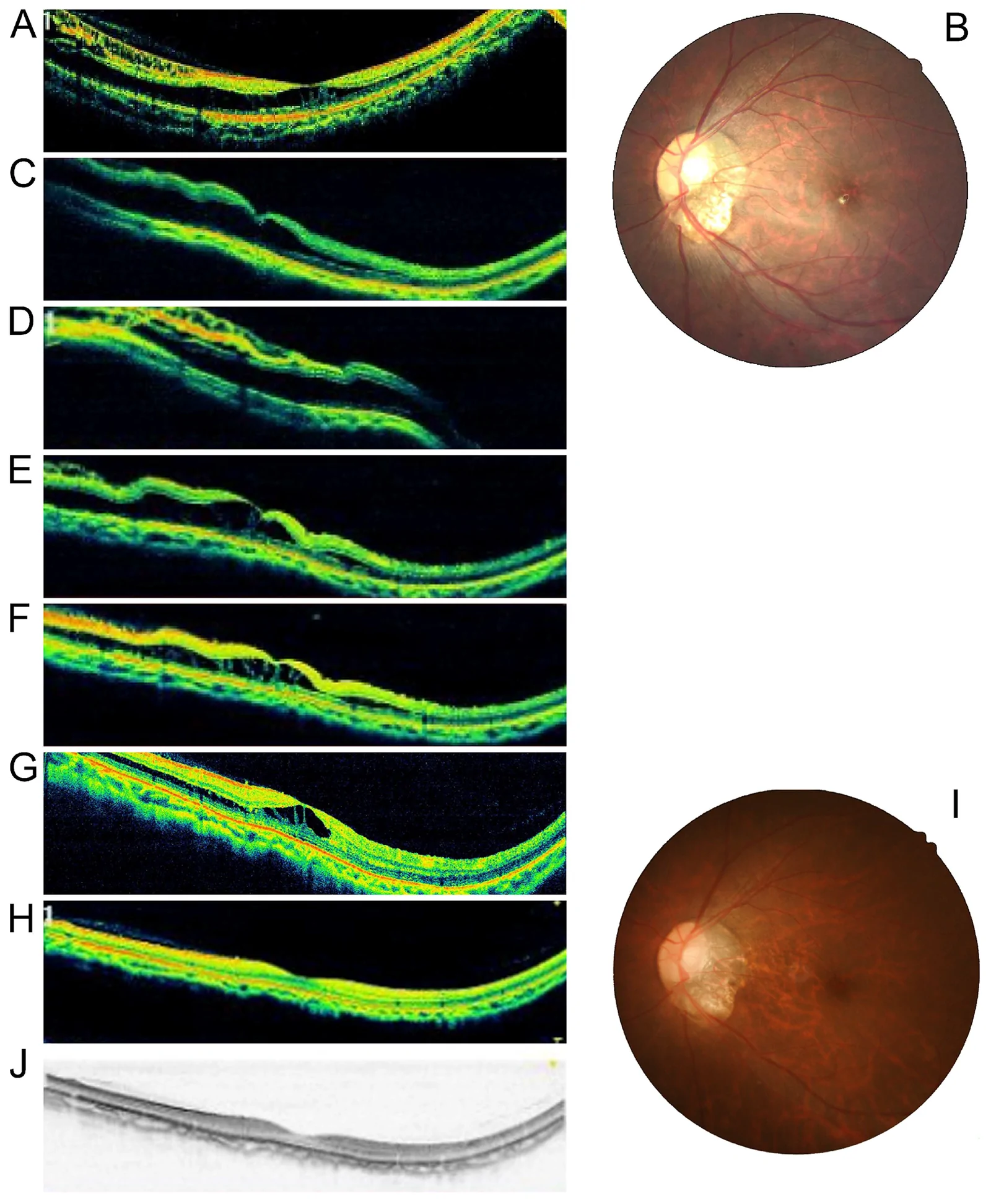
Sequential optical coherence tomography (OCT) and fundus photographs demonstrating resolution of extensive macular retinoschisis (MRS) following posterior scleral contraction (PSC) and scleral strip adjustment (at postoperative day 1) in a 22-year-old female (P19, left eye). **(A)** Preoperative OCT shows MRS involving the fovea; **(B)** Corresponding preoperative fundus photograph reveals a tessellated fundus and temporal crescent; **(C)** On the day following initial PSC, OCT shows persistent MRS with newly formed retinal surface folds; **(D)** On postoperative day 2 (the day after scleral strip adjustment), OCT demonstrates slightly more prominent retinal surface folds; **(E–G)** At 1 week, 1 month, and 6 months postoperatively, OCT images show gradual resolution of MRS and fading of the retinal surface folds; **(H)** At 16 months postoperatively, OCT confirms complete anatomical reattachment; **(I)** Corresponding 16-month fundus photograph shows slight enlargement of the temporal crescent compared to the preoperative state; **(J)** At 31 months (2.5 years) postoperatively, OCT demonstrates maintained structural stability of the macula. Axial length measurements were as follows: preoperative, 27.78 mm; on the day after PSC, 26.15 mm; 1 week postoperative (after band adjustment), 25.38 mm; 2.5 years postoperative, 26.67 mm.

Favorable anatomical outcomes of PSC for MRS are demonstrated in two representative cases. In P1, a 20-year-old male with MRS and macular detachment (Figure 1), OCT showed progressive resolution with complete reattachment achieved by 6 months, and structural stability maintained at the 5-year follow-up. P19, a 22-year-old female with fovea-involving MRS (Figure 2), underwent PSC followed by a secondary scleral strip adjustment. OCT imaging showed gradual resolution of MRS, with complete reattachment by 16 months and maintained stability at the 2.5-year follow-up.

## 4 Discussion

By shortening the axial length, reducing intraocular volume, and relieving vitreoretinal traction, PSC is a promising procedure that facilitates the resolution of MRS and macular detachment (13, 17). By counteracting the tensile forces of staphyloma, PSC can transform the posterior pole contour from convex to a flatter or even slightly concave shape, which reduces localized traction. This mechanism helps alleviate anteroposterior traction induced by the staphyloma and stretched retinal arteries. Properly positioning the crosslinked strip without damaging adjacent ocular structures has been a critical technical challenge in PSC. With nearly two decades of clinical experience, we have refined the surgical technique and modified the allogeneic strip, significantly simplifying the procedure and reducing complication rates (13–16, 18).

MRS is one of the main complications leading to vision loss in highly myopic patients, and it is often asymptomatic at first but gradually worsens over time. MRS may progress to foveal detachment; if left untreated, it can further progress to macular hole or retinal detachment (19). It was reported that timely surgical intervention may be helpful in achieving successful visual outcomes in patients with MRS, and that good baseline BCVA and absence of foveal detachment on OCT preoperatively are important for patient visual prognosis (20). We performed PSC on 21 patients up to age 30 with MRS, and none progressed to macular hole or detachment (*n* = 27). Only three eyes experienced a decrease in BCVA, including one case in which Snellen visual acuity declined from 20/16 to 20/20. This may be related to our occasional practice of stopping measurement at 20/20 without testing for better acuity, and may not represent a true decline in vision. The potential reasons for the decline in BCVA, partial and no resolution of MRS could include: (1) progressive degradation of the implanted strip, and (2) continued axial elongation due to pathologic myopia. Consequently, axial length still regressed toward baseline (Table 1). We believe that adequate intraoperative axial length shortening is critical for resolving significant MRS; therefore, patients with insufficient shortening based on immediate postoperative reassessment underwent secondary scleral strip adjustment in this study. Given the expected natural history of MRS, our findings suggest that PSC is promising in maintaining long-term anatomical stability in patients with MRS up to age 30.

**Table 1:** Comparison of best-corrected visual acuity and axial length, before and after posterior scleral contraction (*n* = 13).

Parameters	Baseline	1 week postop	Approximately 5 years postop	*P-value*
BCVA (LogMAR)	0.10 ± 0.11 (0.10, 0–0.22)	-	0.08 ± 0.10 (0, 0–0.22)	0.482
Axial length (mm)	28.81 ± 1.72	27.39 ± 1.64	28.43 ± 1.77	< 0.001^*^

^*^BCVA, best-corrected visual acuity; LogMAR, logarithm of the minimum angle of resolution.

Regular follow-up is essential for patients with myopic maculopathy, yet the optimal timing for surgical intervention in MTM remains controversial. Although vitrectomy, with or without internal limiting membrane peeling, is effective for MRS (21), it poses unique technical challenges in highly myopic eyes due to excessive axial elongation and posterior staphyloma. Standard vitreoretinal maneuvers, designed for average axial length, may not adequately reach the intended surgical plane in the posterior pole (22). To achieve access to the posterior pole, an indentation of the globe may be created (23), likely causing corneal distortion that compromises the surgical view. Additionally, chorioretinal atrophy, and altered scleral fiber architecture may further compromise surgical outcomes, contributing to difficulties in membrane peeling, postoperative scleral wound leakage and poor anatomical resolution rates. Regarding post-vitrectomy complications, cataract is common, with an incidence reaching 80% within the first postoperative year (24), often necessitating subsequent extraction in younger patients, and cataract surgery following vitrectomy carries a higher risk of zonular dialysis and dropped nuclear fragments compared to routine cases (25). Essentially, vitrectomy does not mitigate the tractional force from posterior staphyloma secondary to axial elongation, so it may not be the optimal surgical choice for younger patients with progressive high myopia and clear lenses.

Compared to vitrectomy, macular buckling has been associated with a higher rate of successful retinal reattachment and a lower risk of MTM progression (26, 27). Furthermore, secondary macular holes developed in 5.4% to 27.3% of cases following vitrectomy for MRS, regardless of whether internal limiting membrane peeling was performed (6, 28, 29). Because factors like vascular traction and posterior staphyloma persist, vitreoretinal surgery alone cannot completely resolve traction maculopathy (30, 31). Besides, longer axial length correlates with higher anatomic failure risk after vitrectomy, attributed to the retina's inability to adapt to progressive axial elongation (32). Macular buckling may alleviate anteroposterior traction induced by staphyloma and vitreous cortex by reshaping the posterior sclera from deeply convex to flat or even concave (33). PSC is also an extraocular procedure, which avoids the inherent risks associated with intraocular surgery. In this study, MRS resolved completely in 30.8% of cases and partially in 69.2% (*n* = 13). The resolution rates are clinically acceptable. Macular condition remained stable over the whole follow-up period. No eyes progressed to macular hole or retinal detachment. The favorable safety profile may be attributable to the extensive experience of the highly specialized surgeon with over 20 years of practice in this procedure. In this study, BCVA improved numerically, but the change did not reach statistical significance, suggesting that the principal benefit of PSC in this study appears to be anatomical stabilization.

Among our patients with MRS treated by PSC, no case of cataract was observed. However, no definitive conclusion can be drawn regarding the absence of cataractogenic risk associated with PSC, given the preliminary nature of this small case series. Metamorphopsia occurred in 12 eyes out of 27 in this study, which may be related to retinal folds secondary to scleral contraction in the early postoperative period (Figures 1, 2). As MRS resolved gradually, the retinal folds flattened out, and metamorphopsia slowly disappeared.

This study has certain limitations. Its retrospective design and inclusion of only those with an approximately 5-year follow-up record may introduce selection bias. The small sample size, though reflective of this specific young cohort, limits generalizability. The absence of a control group, such as observation, vitrectomy or macular buckling, prevents comparative conclusions regarding PSC's efficacy. Finally, outcomes from a single surgeon at a single center may affect reproducibility, and similar results may not be expected outside highly specialized centers.

In conclusion, our findings provide preliminary evidence that early PSC may achieve favorable anatomical stability and an acceptable observed safety profile in young patients with MRS. However, given the inherent limitations of the study design, these results should be interpreted with caution. Further prospective, controlled studies with larger sample sizes and longer follow-up are warranted to confirm the long-term efficacy and safety of this procedure.

## Data Availability

The raw data supporting the conclusions of this article will be made available by the authors, without undue reservation.
